# Targeting TGF-β Signaling in Kidney Fibrosis

**DOI:** 10.3390/ijms19092532

**Published:** 2018-08-27

**Authors:** Yoshitaka Isaka

**Affiliations:** Department of Nephrology, Osaka University Graduate School of Medicine, Suita 565-0871, Japan; isaka@kid.med.osaka-u.ac.jp; Tel.: +81-6-6879-3857; Fax: +81-6-6879-3230

**Keywords:** glomerulosclerosis, interstitial fibrosis, antisense, siRNA, gene therapy, pirfenidone, ligand trap, galunisertib

## Abstract

Renal fibrosis is the final common pathway of numerous progressive kidney diseases, and transforming growth factor-β (TGF-β) has an important role in tissue fibrosis by up-regulating matrix protein synthesis, inhibiting matrix degradation, and altering cell-cell interaction. Many strategies targeting TGF-β, including inhibition of production, activation, binding to the receptor, and intracellular signaling, have been developed. Some of them were examined in clinical studies against kidney fibrosis, and some are applied to other fibrotic diseases or cancer. Here, I review the approaches targeting TGF-β signaling in kidney fibrosis.

## 1. TGF-β as a Target for Kidney Fibrosis

Renal fibrosis, the final common pathway of numerous progressive kidney diseases, is characterized by an increase in extracellular matrix (ECM) protein production, a decrease in matrix degradation, dysregulation of cell-matrix interaction, inflammatory cell infiltration, and transformation of resident cells. Transforming growth factor-β (TGF-β), a multifunctional dimeric peptide, regulates biological processes such as cell proliferation, differentiation and immunological reaction. One of the most important biological actions of TGF-β is the regulation of ECM accumulation [1]. TGF-β up-regulates the synthesis of individual matrix components, including collagens, proteoglycans, and glycoproteins [2]. Conditioned media from cultured nephritic glomeruli induced elevated proteoglycan synthesis in normal mesangial cells, and this response was mimicked by exogenous TGF-β, but was blocked by addition of TGF-β antiserum. TGF-β also inhibits matrix degradation by decreasing the synthesis of metalloproteinases and increasing the synthesis of inhibitors of metalloproteinases [3]. Nephritic glomeruli exhibited the suppressed plasminogen activator (PA) and up-regulated plasminogen activator inhibitor-1 (PAI-1). Addition of TGF-β to normal glomeruli markedly reduced the activity of PA and dramatically increased the synthesis of PAI-1 [3]. TGF-β alters the integrin expression and modulates their relative proportions on the cell surface in a fashion that can facilitate adhesion to the matrix [4]. In nephritic glomeruli, mesangial expression of α1β1 and α5β1 integrins and their ligands (e.g., laminin, collagen and fibronectin) were paralleled with TGF-β1 protein expression. TGF-β also induces the transformation of resident cells [5]. TGF-β has been shown to activate the pericyte–myofibroblast transition of tubular epithelial cells in an obstructive kidney fibrosis model, and this phenotypic alteration was blocked by anti-TGF-β1 antibody or TGF-β type I receptor inhibitor [5].

A number of evidences, including up-regulation of TGF-β signaling in glomeruli or tubulointerstitium in fibrotic kidney, kidney fibrosis-induced by increased TGF-β, and amelioration of kidney fibrosis by anti-TGF-β therapy, have demonstrated the direct TGF-β actions in fibrotic kidney diseases. In human glomerular diseases, an increased TGF-β expression was observed in progressive glomerular diseases and fibrotic areas were strongly correlated with TGF-β1 expression in biopsy specimens [6]. Glomeruli from minimal change nephritic syndrome with minor glomerular abnormalities, showed weak immunostaining for TGF-β, which was similar to normal kidneys. Conversely, kidneys from diabetic nephropathy, Immunoglobulin A (IgA) nephropathy, focal and segmental glomerulosclerosis (FSGS), crescentic glomerulonephritis, and lupus nephritis showed significantly increased expression of all three TGF-β isoforms in glomeruli and tubulointerstitium [7]. *TGF-β1* mRNA expression in glomeruli with moderate mesangial matrix deposition was higher than that in normal glomeruli [8]. The expression of TGF-β receptors was also up-regulated in diseased glomeruli. In minor glomerular abnormalities, classical TGF-β type I receptor (activin receptor-like kinase 5; ALK5) and TGF-β type II receptor (TGFβRI and TGFβRII, respectively) were weakly expressed mainly in glomerular endothelial cells, peritubular capillaries, and interstitial arteriolar endothelial cells [9]. On the contrary, TGFβRI and TGFβRII were increased remarkably in glomeruli with increased matrix accumulation in renal biopsy specimens from nephritic patients [10]. Sustained or excessive expression of TGF-β is considered to be a major cause of glomerular matrix expansion in human and experimental glomerular diseases [11,12]. Diabetic rats exhibited a slow, but progressive increase in TGF-β mRNA and protein expression in glomeruli, and diabetic nephropathy patients also showed increased immunoreactive TGF-β protein with concomitant glomeruli deposition of fibronectin extra domain A isoform (FnEDA) [7]. In the progressive fibrotic kidney disease model by repeated injection of anti-mesangial serum, the glomerular expression of *TGF-β1* mRNA and TGF-β1 protein remained elevated, which was associated with glomerulosclerosis and tubulointerstitial fibrosis with marked deposition of collagens type I and III [12]. In this progressive kidney fibrosis model, sustained glomerular TGF-β1 was suggested to activate tubulointerstitial cells to produce TGF-β1, leading to ECM deposition in the interstitium. It was hypothesized that a failure to shut down TGF-β due to TGF-β dysregulation or repeated insults may lead to a vicious cycle of sustained production of TGF-β and ECM [12]. The pathogenesis of diabetic kidney diseases was also associated with the increased renal expression of TGF-β [7,13].

Increased exogenous or intrarenal TGF-β was demonstrated to induce fibrotic manifestation in the kidneys as follows. Sharma et al. examined the immunoreactive TGF-β content in renal blood and urine, and demonstrated that diabetic patients produced TGF-β in their kidneys, but that non-diabetic patients extracted circulating TGF-β from their kidneys, suggesting that increased renal TGF-β production may be an important manifestation of diabetic kidney disease [13]. Intravascular injection of TGF-β into rats produced fibrotic lesions in multiple target tissues, including the liver, bone, kidney, heart, and so on. In the kidneys, TGF-β administration rapidly caused glomerulosclerosis [14]. *TGF-β1* gene transfection via the renal artery induced glomerular mesangial overexpression of *TGF-β1*, leading to glomerulosclerosis within several days without immunological insults [15]. *TGF-β1*-transfected kidneys exhibited extensive type I and type III collagen deposition with mild mesangial proliferation. Hepatic overexpression of *TGF-β* resulted in renal fibrosis as well as hepatic fibrosis [16]. *TGF-β1* transgenic mice, under the control of the murine albumin promoter, express the transgene exclusively in the liver and have elevated plasma concentrations of TGF-β1. These mice showed mesangial expansion and thickened capillary loops at three weeks, and subsequently exhibited interstitial fibrosis and tubular atrophy. Two of the three lines of these transgenic mice, which had the highest levels of hepatic transgene expression and the highest plasma *TGF-β1* levels, exhibited renal manifestations. Overexpression of *TGF-β* in tubular epithelial cells directly induced interstitial fibrosis without injury [17]. The transgenic mice with the highest levels of *TGF-β1* developed proteinuria by five weeks of age. These mice subsequently manifested nephrotic syndrome with ascites and progressive azotemia by 15 weeks of age. Increased levels of circulating *TGF-β1* induced progressive renal disease that was characterized by mesangial expansion, accumulation of glomerular immune deposits and matrix proteins, and interstitial fibrosis in this transgenic mouse model. Renal tubular cell-specific *TGF-β1* transgenic mice showed widespread peritubular fibrosis and focal degeneration of nephrons. Tubular cell-derived TGF-β1 induced robust proliferation of peritubular cells and deposition of collagen, leading to the degeneration of nephrons in a focal pattern via TGF-β1-driven autophagy.

Contrarily, anti-TGF-β treatment abrogated the kidney fibrosis as follows. Renal fibrosis has been blocked by in vivo injection of anti-TGF-β neutralizing antibodies [18] and anti-TGF-β type II receptor (TGFβRII) antibodies [19]. Administration of anti-TGF-β1 at the time of induction of the acute mesangial proliferative glomerulonephritis, which is associated with increased production and activity of TGF-β1, suppresses the increased production of ECM and dramatically attenuates histological manifestations of the disease [18]. TGFβRII was up-regulated in mesangial proliferative lesions, tubular cells and interstitial cells in the experimental mesangial proliferative model rats. Treatment with antibody against TGFβRII also suppressed mesangial matrix expansion, with decreased urinary protein excretion, compared with control mesangial proliferative glomerulonephritis model rats [19]. Furthermore, inhibition of *TGF-β* gene expression by antisense oligodeoxynucleotides (ODNs) could ameliorate fibrotic manifestation in experimental glomerulonephritis [20] and diabetic animal models [21,22], as well as interstitial fibrosis in unilateral ureteral obstruction model [23]. These evidences strongly suggest that the inhibition of TGF-β action should be viable therapeutic strategies to prevent the progression of renal fibrosis.

## 2. Clinical Trials Targeting TGF-β

Several clinical studies targeting TGF-β for fibrotic kidney diseases, as well as other fibrotic diseases or cancer, have been reported (Table 1). A randomized, double-blind, phase II trial was performed using a humanized neutralizing monoclonal antibody against TGF-β1 (LY2382770) for the treatment of diabetic nephropathy patients (NCT01113801) [24]. 416 randomized patients with type 1 or type 2 diabetes, a serum creatinine (SCr) level of 1.3–3.3 mg/dL for women and 1.5–3.5 mg/dL for men, and a 24-h urine protein-to-creatinine ratio (UPCR) >800 mg/g, received monthly anti-TGF-β1 monoclonal antibody (2, 10 or 50 mg) or placebo subcutaneously for 12 months to assess a change in SCr (primary outcome). In this study, no safety issues were observed. The overall frequencies of the adverse events, of which there were several types including end stage kidney disease, acute kidney injury, and death, were not different between the TGF-β1 antibody-treated group and placebo-treated group. However, this trial was prematurely ended for its futility. This study did not show efficacy of the anti-TGF-β1 antibody on SCr changes from the baseline, estimated glomerular filtration rate (eGFR), and UPCR, over any of the three doses. The percent change in SCr from the baseline to end point was not different between the placebo group (14%; 95% confidence interval (CI) 9.7–18.2%) and the anti-TGF-β1 antibody-treated group at 2 mg (20%; 95% CI 15.3–24.3%), 10 mg (19%; 95% CI 14.2–23.0%) and 50 mg (19%; 95% CI 14.0–23.3%). Treatment with placebo or anti-TGF-β1 antibody had no apparent effect on the change in proteinuria from the baseline to the 6 month or end point of the trial.

A randomized, double-blind, phase I and II trial were performed using fresolimumab, a humanized monoclonal antibody, against all three isoforms of TGF-β for the treatment of FSGS patients [25,26]. The phase I clinical trial of fresolimumab on FSGS patients demonstrated that a single dose of infusion of fresolimumab up to 4 mg/kg was safe and well-tolerated in FSGS patients, and that the half-life of fresolimumab was 14 days [26]. In the phase II clinical trial, steroid-resistant FSGS patients received 1 or 4 mg/kg of fresolimumab or placebo at day 1, 28, 56, and 84, and were followed in a double-blind way for 252 days. The primary efficacy endpoint was the percentage of patients achieving partial (50% reduction) or complete (<300 mg/g Cr) remission of proteinuria. This study was also closed before reaching the target number of patients. At day 112, the mean percent change in UPCR was −18.5% (*p* = 0.008), +10.5% (*p* = 0.52), and +9.0% (*p* = 0.91) in patients treated with 1 mg/kg, 4 mg/kg of fresolimumab, and placebo, respectively. A nonsignificant but greater eGFR decline in the placebo group was observed, compared with the fresolimumab groups. Although an eGFR stabilization trend was observed, it was not clearly related to the degree of the proteinuria reduction, suggesting that the effect of fresolimumab in retarding the progression of kidney fibrosis—and thereby eGFR stabilization—may have been independent of glomerular permeability improvement. Although these two studies failed to demonstrate the therapeutic efficacy of systemic administration of anti-TGF-β antibody on fibrotic kidney diseases, they have several limitations which confound drawing definitive conclusions that anti-TGF-β antibody treatment is not useful against fibrotic kidney diseases. If less severe- or earlier staged-patients were enrolled, anti-TGF-β antibody treatment may have been effective. Besides, more complete TGF-β inhibition might be necessary to retard the progression of fibrotic kidney disease. From this point of view, it is possible that a pharmacologically relevant amount of systemically-administered anti-TGF-β antibody may not reach the kidney’s fibrotic site. Recently, targeting anti-TGF-β antibody therapy to fibrotic kidneys using a dual specificity approach was reported [27]. Of interest is that this antibody was designed to reach the TGF-β-specific ECM protein, FnEDA. This antibody is a dual specific antibody with a moiety to target FnEDA and a second moiety to neutralize TGF-β. Thus, systemically-administered- dual specific antibody was found to accumulate highly in the fibrotic area of obstructed kidneys, but not in non-obstructed kidneys. By contrast, systemically-administered monoclonal anti-TGF-β antibody distributed in lower concentration in the obstructed kidneys as well as in non-obstructed kidney. Although the dual specific antibody and the simple monoclonal anti-TGF-β antibody similarly reduced the progression of fibrosis in obstructed kidneys, the advantage of the dual specific antibody is that it does not interfere with the TGF-β activity in the non-fibrotic area. It is possible that a systemically-administered antibody may affect the complex and pleiotropic TGF-β functions, for example, wound healing, tissue regeneration, and anti-inflammatory actions. Therefore, fibrotic area-targeting therapy, such as dual specific antibody, may enable us to inhibit TGF-β completely by dosing up to a maximum range without side effects.

Pirfenidone, which has been shown to reduce TGF-β1 production, is an orally available small molecule. Pirfenidone has been shown to suppress *TGF-β* gene expression at the transcription level [28], and also suppress TGF-β protein expression [29]. The therapeutic function of pirfenidone was examined in animal models with pulmonary fibrosis or kidney fibrosis, wherein Bleomycin was used to induce TGF-β overexpression and lung fibrosis. Treatment with pirfenidone in the bleomycin-induced lung fibrosis model suppressed *TGF-β* gene transcription by 33% [28]. Chronic cyclosporine (CsA) nephrotoxicity is characterized by tubulointerstitial fibrosis with increased TGF-β. Administration of pirfenidone suppressed TGF-β1 protein expression by 80%, and ameliorated CsA-induced fibrosis by about 50% [29]. Pirfenidone also inhibited fibrosis and eGFR decline in the kidney disease model [30]. Clinical trials [31] have evaluated the safety and efficacy of pirfenidone in various fibrotic diseases, including pulmonary fibrosis. Pirfenidone reduced disease progression, assessed by lung function, exercise tolerance, and progression-free survival in idiopathic pulmonary fibrosis patients compared to placebo-treated patients. Gastrointestinal and skin-related adverse events were more common in the pirfenidone group than in the placebo group, but the side-effects were almost acceptable [31]. Against fibrotic kidney diseases, an open-label trial was performed to evaluate the safety and efficacy of pirfenidone on FSGS patients [32]. Adverse events associated with pirfenidone therapy were dyspepsia, abdominal discomfort, sedation or fatigue, and so on. Pirfenidone had no effect on blood pressure (BP) or proteinuria. Proteinuria was 3.2 g/day (interquartile range (IQR) 1.6 to 4.4) at baseline, and 4.3 g/day (IQR 1.2 to 6.7) at the 12-month study visit (*p* = 0.16). In contrast to the effect on proteinuria, pirfenidone slowed the monthly change in eGFR. During the baseline period, the monthly change in eGFR was −0.61 mL/min/1.73 m^2^ (IQR −1.31 to −0.41), but improved to −0.45 mL/min/1.73 m^2^ (IQR −0.78 to −0.16) during the treatment period [32]. A randomized, double-blind, placebo-controlled study was also performed to evaluate the effect of pirfenidone on diabetic nephropathy patients with elevated albuminuria and reduced eGFR [33]. There were no significant differences in albuminuria among study groups from baseline to the end of study (*p* = 0.19). Of interest is that urinary TGF-β excretion was increased by 1.4 pg/mg creatinine (95% CI; 0.2–6.6) over 12 months in the placebo group, increased by 0.3 pg/mg (95% CI; −1.6–5.6) in the 1200 mg of pirfenidone group, and decreased by 0.1 pg/mg (95% CI; −3.4–3.7) in the 2400 mg of pirfenidone group (*p* = 0.54). This study demonstrated that the eGFR mean increased in the pirfenidone group (+3.3 ± 8.5 mL/min/1.73 m²), but decreased in the placebo group (−2.2 ± 4.8 mL/min/1.73 m²; *p* = 0.026). In these studies, pirfenidone failed to have a beneficial effect on proteinuria, but may have ameliorated fibrosis in the tubulointerstitium without affecting glomerular injury, although the mechanism was not clear. Taking these results together, anti-TGF-β therapy might exert its beneficial effects mainly by reducing fibrosis in the tubulointerstitium, rather than causing glomerular changes. A previous animal study demonstrated that treatment with an anti-TGF-β antibody did not affect proteinuria, but improved other functional parameters, including glomerular matrix expansion and renal function in a diabetic nephropathy model [21]. Short-term anti-TGF-β treatment may not improve macromolecular permeability in diabetes. However, *TGF-β1* transgenic mice developed glomerular basement membrane thickening and proteinuria [16]. Therefore, a long-term and continuous complete blockade of glomerular TGF-β may reduce proteinuria.

## 3. Future Strategy Targeting TGF-β

**Figure 1:**
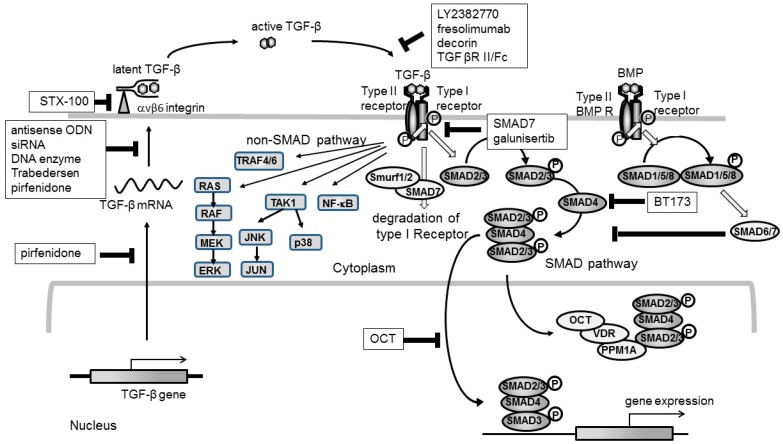
Schematic overview of the strategies targeting TGF-β signaling. The first step is the inhibition of production of TGF-β at the transcription and translation level. Transcription or translation of *TGF-β* mRNA can be blocked by pirfenidone, antisense oligodeoxynucleotides (ODN) (Trabedersen), siRNA, or DNA enzymes. In this step, we should develop a vehicle to carry the drug to the inside of the specific cells. The second step is inhibitory strategies targeting the activation of TGF-β, and its binding to the receptor. Integrin αvβ6 activates TGF-β, and anti-αvβ6 antibody (STX-100) is expected to suppress kidney fibrosis by antagonizing the activation of TGF-β. A ligand trap to block the binding of active TGF-β to its receptor was clinically examined by using the antibody for TGF-β (LY2382770, fresolimumab). Decorin or TGFβRII/Fc was reported to competitively suppress TGF-β-induced fibrosis in an animal model. The third step is the blockade of intracellular signaling following the activation of the TGF-β receptor. Galunisertib is an oral inhibitor of TGF-β type I receptor kinase, and down-regulates the phosphorylation of SMAD2/3. SMAD6/7 can inhibit the phosphorylation of SMAD2/3, and thereby block TGF-β signaling. Additionally, SMAD7 degrades type I TGF-β receptor in combination with Smurf1/2. BT173, an inhibitor of homeo-domain interacting protein kinase 2 (HIPK2), which binds to HIPK2 and reduces the interaction between HIPK2 and SMAD4, thereby inhibiting the subsequent SMAD3 phosphorylation and activation. 22-oxacalcitriol (OCT) binds to the vitamin D receptor (VDR), forming a PPM1A/VDR/OCT complex, which dephosphorylates SMAD3, terminating TGF-β-SMAD3 signaling. BMP-SMAD1/5/8 signaling antagonize TGF-β-SMAD3 signaling by inducing SMAD6/7. (Grey arrow means promotion, and T bar means inhibitory effect.).

Many strategies targeting TGF-β signaling have been developed, some of which have reached clinical studies (Figure 1). The reduction of TGF-β synthesis is one of the strategies to reduce excessive or sustained TGF-β expression in the kidneys. In animal models, several methods to reduce TGF-β expression in mesangial cells and interstitial cells have been reported by using antisense ODNs [17,34], DNA enzymes [35,36], and small interfering RNAs (siRNAs) [37]. Introduction of TGF-β antisense ODNs into mesangial cells of mesangioproliferative nephritic rats [17] or interstitial fibroblasts of ureteral obstructed kidneys [38], using the hemagglutinating virus of Japan (HVJ)-liposome method, has been shown to inhibit TGF-β expression in glomeruli or interstitium, consequently suppressing glomerular or interstitial fibrosis, respectively. We also demonstrated that introduction of a DNAzyme for TGF-β into glomerular mesangial cells by electroporation-mediated gene transfer suppresses TGF-β expression and thereby inhibits consequent glomerular ECM accumulation in anti-Thy-1 nephritic kidneys [36]. Furthermore, electroporation-mediated introduction of siRNA into mesangial cells, targeting against TGF-β1, has been shown to significantly suppress TGF-β1 mRNA and protein expression, thereby ameliorated glomerular matrix expansion in experimental glomerulonephritis [37].

These strategies to reduce TGF-β synthesis have several limitations for clinical application. The most important hurdle in kidney diseases is the introduction of relatively large molecules to target cells, e.g., mesangial cells, tubular epithelial cells or interstitial cells. The success of performing gene therapy depends on the selection of an approach to target cells, and the transfection tool. In the former step, various approaches, such as systemically or directly via renal artery, renal vein, or ureter, are available to deliver genes to the kidney cells. These approaches may also determine the transduced cells, such as glomerular cells, tubular cells or interstitial fibroblasts. A transfer tool may be used to recognize target cells to effectively introduce genes into a particular cell. Conjugating peptides or antibodies, binding to a specific surface antigen, increases the efficacy of transduction. Recently, it was reported that an adenoviral vector with specific promotors for each of the tubular cells, such as sodium-dependent phosphate transporter type 2a for proximal tubules, sodium-potassium-2-chloride cotransporter for the thick ascending limb of Henle, and aquaporin 2 for the collecting duct, succeeded in cell-specific transduction by administration via the renal artery [39]. In the latter step to introduce genes into cells, the selection of a viral or non-viral vector affected the transfection efficacy. For the clinical application of gene therapy against kidney fibrosis targeting TGF-β, we need to introduce genetic material into mesangial cells in patients with glomerulosclerosis, or interstitial fibroblasts in patients with interstitial fibrosis. To date, we have not succeeded in developing a method to introduce genetic material effectively to kidney cells. Therefore, clinical studies for gene therapy against fibrotic kidney diseases have not been reported. In a clinical study for high grade glioma, however, antisense ODNs against-β2 (trabedersen, AP 12009) were reported to be superior and safer compared with standard chemotherapy, as follows [40]. In this randomized, open-label, active-controlled, dose-finding phase IIb study, the researchers were examining the efficiency and safety of trabedersen, which recurrent/refractory high-grade glioma patients received intratumorally. Prespecified anaplastic astrocytoma subgroup analysis demonstrated a significant benefit on the 14-month tumor control rate for 10 µM trabedersen vs. chemotherapy (*p* = 0.0032) [40]. The two-year survival rate had a trend for superiority for 10 µM trabedersen vs. chemotherapy (*p* = 0.10). The frequency of patients with related or possibly drug-related adverse events was higher with standard chemotherapy (64%) than with 80 µM trabedersen (43%) and 10 µM trabedersen (27%) [40]. Therefore, this vehicle development to deliver genetic materials to specific cells may benefit the development of gene therapies targeting TGF-β against kidney fibrosis.

The next strategy is to suppress the activation TGF-β. Newly synthesized TGF-β is secreted in a biologically inactive latent form by binding to the latency-associated peptide (LAP), and is anchored to ECM. Exposure to several factors, including reactive oxygen species (ROS) and integrin αvβ6 [41] activates TGF-β. In the kidneys, β6 integrin was expressed in tubular epithelial cells, but not in glomeruli, suggesting that αvβ6-mediated TGF-β activation has an important role in tubulointerstitial fibrosis. Integrin β6-null mice showed less active TGF-β protein expression with lower collagen content in a unilateral ureteral obstruction (UUO) model compared with wild type mice, despite robust macrophage infiltration [42]. The effect of anti-αvβ6 integrin antibody was tested in lung fibrosis, because integrin αvβ6 was overexpressed in lung pheumocytes lining the alveolar ducts and alveoli in idiopathic pulmonary fibrosis patients. A low dose of anti-αvβ6 antibody was demonstrated to partially block TGF-β activity and attenuate collagen expression without increasing the alveolar inflammatory cell population or macrophage activation in a mouse pulmonary fibrosis model [43]. However, a high dose of anti-αvβ6 antibody increased expression of markers of inflammation and macrophage activation, with the inhibitory effect on TGF-β [44]. A phase II clinical study using anti-αvβ6 antibody (STX-100) is now underway in interstitial pulmonary fibrosis patients (NCT01371305). Besides αvβ6 integrin, other mechanisms also activate TGF-β. Thus, it is possible that the treatment with anti-αvβ6 antibody can partially block the activities of TGF-β.

Another strategy is the ligand trap to inhibit the binding of the activated TGF-β to its receptors. Activated TGF-β can bind to the cell surface receptor, TGF-β receptor type I and type II. The active TGF-β1 and TGF-β3 directly bind type II receptors in the absence of a type I receptor, whereas TGF-β2 binding requires both type I and type II receptors [44]. The type II receptors recognize the active TGF-β1 ligand. Although an inactive soluble type II receptor (monomer) may impede the TGF-β1 activities as a ligand trap, it was shown to have approximately 10-fold lower binding affinity for TGF-β1 than cell-surface type II receptor [45]. Compared with a monomer type II receptor, a chimeric molecule termed TGFβRII/Fc (the extracellular portion of the TGF-β type II receptor fused to an immunoglobulin heavy-chain Fc fragment) effectively blocked the binding of TGF-β1 to the cell surface receptor [46]. Therefore, treatment with TGFβRII/Fc has been shown to suppress ECM production for proliferative glomerulonephritis in model rats [46]. TGFβRII/Fc was reported to bind TGF-β1 and TGF-β3, but not TGF-β2 [47]. While all three TGF-β isoforms have been shown to induce the ECM protein, only TGF-β2 has been shown to have anti-fibrotic effects [48]. Thus, TGFβRII/Fc may be useful for selective blockade of TGFβ1/3 in kidney fibrosis. One of the matrix components induced by TGF-β, the proteoglycan decorin, can also bind TGF-β and neutralize its biological activity. Administration of decorin [49] or gene transfer of the decorin gene into muscle [50] inhibited the increased ECM production and attenuated manifestations of acute mesangial proliferative glomerulonephritis in model rats via the ligand trap, by binding of the protein to TGF-β receptors.

Interaction of TGF-β with the receptors results in several cellular responses by SMAD- and non-SMAD-dependent pathways [51]. There are two ligand TGF-β superfamilies: TGF-β-Activin-Nodal and the BMP. SMAD2 and SMAD3 are activated by TGF-β, whereas SMAD1, SMAD5 and SMAD8 are activated by BMP. TGF-β-SMAD2/3 signaling and BMP-SMAD1/5/8 signaling antagonize each other by inducing antagonistic factors, including SMAD6/7. BMP7 was demonstrated to have a protective function in kidney fibrosis by antagonizing TGF-β1′s action via anti-inflammatory, anti-oxidant, and anti-fibrotic functions [52]. Additionally, TGF-β signaling is mediated via non-SMAD molecules, including tumor necrosis factor (TNF) receptor-associated factor 4 (TRAF4), TRAF6, TGF-β-activated kinase 1 (TAK1), p38 mitogen-activated protein kinases (MAPKs), extracellular signal-regulated kinase (ERK), c-JUN N-terminal kinase (JNK), RAS (rat sarcoma)-RAF (rapidly Accelerated fibrosarcoma)-activating MAPK/ERK kinase (MEK) and ERK, and nuclear factor-κB (NF-κB). TGF-β-induced activation of the ERK and JNK pathway can result in SMAD phosphorylation and regulate SMAD activation [51]. In addition, TGF-β-induced RAS/ extracellular signal-regulated kinase (ERK) MAPK signaling induces TGF-β1, and amplifies TGF-β responses [53]. Although non-SMAD pathways have been reported to mediate TGF-β-induced fibrosis [54], TGF-β develops fibrosis mainly via SMAD signaling [55].

In the SMAD-dependent pathway, binding of the TGF-β type II receptor allows its dimerization with the type I receptor, and induces transphosphorylation in the type I receptor by the type II receptor kinases. Consequently, activated type I receptor phosphorylates SMAD2/3, then form a complex with SMAD4. Activated SMAD complexes translocate into the nucleus, where they regulate the transcription of the target genes. Several strategies have been attempted to target the above TGF-β signaling pathway, as follows. Galunisertib (LY2157299) is an oral inhibitor of TGF-β type I receptor kinase, and down-regulates the phosphorylation of SMAD2/3. The clinical trial to test the efficacy of galunisertib on cancer may also be applicable to kidney fibrotic diseases, because the anti-fibrotic potency of galunisertib was reported in a liver fibrotic model [56]. In this model, galunisertib inhibited the phosphorylation of SMAD2/3, but did not change the phosphorylation of SMAD1, thereby suppressing the production and maturation of collagen [56]. Of interest is that conditional deletion of SMAD2 enhanced renal fibrosis, because SMAD2 deficiency induced excess phosphorylation of SMAD3, resulting in the SMAD3 binding to a collagen promotor and auto-induction of TGF-β [56]. Conversely, SMAD2 overexpression inhibited TGF-β1-induced phosphorylation of SMAD3, as well as type I collagen expression in tubular cells [56].

SMAD6/7 can inhibit the phosphorylation of SMAD2/3, thereby blocking TGF-β signaling. Additionally, SMAD7 was demonstrated to down-regulate TGF-β signaling via recruiting E3 ubiquitin ligase SMAD-ubiquitination-regulatory factor 1 (Smurf1) and Smurf2, which degrade the type I TGF-β receptor through a proteasomal and lysosomal pathway [10,57]. TRAF6 was also associated to regulate intramembrane proteolytic cleavage of TGF-β type I receptor in cancer cells, where the intracellular domain (ICD) of the TGF-β type I receptor enters into the nucleus, thereby activating genes involved in tumor cell invasiveness, including matrix metalloproteinase2 (MMP2) and Snail [58]. Cleavage requires the combined activities of the TRAF6 and TNF-α-converting enzyme (TACE). This cleavage event was reported to occur selectively in cancer cells [59]. It is possible that this cleavage of the type I receptor is important in TGF-β-mediated tumor invasion, but not in the fibrotic process. Smurf1/2 also antagonize TGF-β signaling by interacting with SMAD2/3 to degrade them [60]. SMAD7 suppressed SMAD signaling from all the TGF-β superfamily, whereas SMAD6 is a specific inhibitor of the BMP signaling pathway. It was reported that overexpression of SMAD7 inhibited renal fibrosis in diabetic kidney [56] and in an obstructed kidney model [56], while a *SMAD7* gene deletion promoted renal fibrosis [56]. Thus, SMAD7 seems to be a promising treatment, if a method for efficacy of introducing *SMAD7* gene into mesangial cells or interstitial fibroblasts is developed.

Another target molecule to inhibit the TGF-β-SMAD pathway is homeo-domain interacting protein kinase 2 (HIPK2), which regulates gene expression by phosphorylating transcription factors and accessory components, and induces apoptosis and epithelial-to-mesenchymal transition markers by physical association with SMAD3 to modulate the activity of TGF-β-SMAD3 signals. It was up-regulated in kidneys from human immunodeficiency virus (HIV) transgenic mice and patients with HIV nephropathy and FSGS [61]. An inhibitor of HIPK2, BT173, ameliorated kidney fibrosis via inhibiting the TGF-β1-SMAD3 pathway [62]. BT173 binds to HIPK2 and reduces the interaction between HIPK2 and SMAD4, thereby inhibiting the subsequent SMAD3 phosphorylation and activation [62].

Unlike the temporary expression of TGF-β in tissue repair, sustained TGF-β expression leads to kidney fibrosis. Thus, termination of TGF-β is ideal. Protein phosphatase Mg^2+^/Mn^2+^-dependent 1A (PPM1A) was revealed to dephosphorylate SMAD2/3, and terminate TGF-β1 stimulation [63]. Under the stimulation of TGF-β, nuclear PPM1A physically interacted with the vitamin D receptor (VDR), and the PPM1A/VDR complex was not recruited to phosphorylated SMAD3. On the contrary, treatment with 22-oxacalcitriol (OCT), which bound to VDR, and formed PPM1A/VDR/OCT complex, dephosphorylated SMAD3, and terminated TGF-β-SMAD3 signaling [64].

## 4. Side Effect of Targeting TGF-β

It is possible that continuous suppression of TGF-β function may lead to unacceptable toxicity, because TGF-β has a highly pleiotropic function. Although it was reported that lifetime blocking of TGF-β activity did not cause any apparent adverse side effects in soluble TGF-β receptor IgGFc chimera transgenic mice [56], long-term treatment with systemic TGF-β suppression may affect wound healing, tissue repair, and anti-inflammatory actions. It has been suggested that systemic shutdown of the TGF-β activities may ameliorate its fibrotic effects, but also block its anti-inflammatory functions. In fact, a high dose of anti-αvβ6 antibody was reported to induce inflammation and macrophage activation with the inhibitory effect on TGF-β [43]. It was also shown that conditional knockout of TGF-βRII in renal tubular cells exhibited increased renal inflammation with up-regulation of interleukin-1β (IL-1β) and tumor necrosis factor-α (TNF-α) after induction of ureteral obstruction [65]. Long-term treatment with anti-TGF-β inhibitors may be ill-advised even in cancer therapy [66]. The results obtained from preclinical animal studies are expected to translate to human clinical studies. We should examine more optimal strategies to block the function of TGF-β in fibrotic kidney, that minimally interfere in other organs. To overcome these adverse events, methods for site-specific TGF-β blockade or shutdown of the vicious cycle of TGF-β signaling should be developed.

## Figures and Tables

**Table 1:** Clinical trials targeting transforming growth factor-β (TGF-β).

Drug	Target	Disease	Results	Reference
LY2382770	TGF-β1	diabetic nephropathy	No efficacy on change in SCr, eGFR, and proteinuria	[24]
fresolimumab	TGF-β1,2,3	FSGS	less eGFR decline (not significant)	[25,26]
pirfenidone	TGF-β1,2,3	FSGS	slower eGFR decline, no effect on BP or proteinuria	[30]
		diabetic nephropathy	Increased eGFR	[32]
		pulmonary fibrosis	reduced disease progression and death	[33]
STX-100	αvβ6 integrin	pulmonary fibrosis	Ongoing phase 2 trial (NCT01371305)	
galunisertib	Type I receptor kinase	cancer	Ongoing clinical trial on glioblastoma, pancreatic cancer, and so on	
trabedersen	TGF-β2	glioma	Superiority of tumor control and survival over chemotherapy	[40]

## References

[1] Border W., Noble N. (1994). Transforming growth factor in tissue fibrosis. N. Engl. J. Med..

[2] Okuda S., Languino L.R., Ruoslahti E., Border W.A. (1990). Elevated expression of transforming growth factor-β and proteoglycan production in experimental glomerulonephritis. Possible role in expansion of the mesangial extracellular matrix. J. Clin. Investig..

[3] Tomooka S., Border W.A., Marshall B.C., Noble N.A. (1992). Glomerular matrix accumulation is linked to inhibition of the plasmin protease system. Kidney Int..

[4] Kagami S., Border W.A., Ruoslahti E., Noble N.A. (1993). Coordinated expression of β1 integrins and transforming growth factor-β-induced matrix proteins in glomerulonephritis. Lab. Investig..

[5] Wu C.F., Chiang W.C., Lai C.F., Chang F.C., Chen Y.T., Chou Y.H., Wu T.H., Linn G.R., Ling H., Wu K.-D. (2013). Transforming growth factor β-1 stimulates profibrotic epithelial signaling to activate pericyte-myofibroblast transition in obstructive kidney fibrosis. Am. J. Pathol..

[6] Yoshioka K., Takemura T., Murakami K., Okada M., Hino S., Miyamoto H., Maki S. (1993). Transforming growth factor-β protein and mrna in glomeruli in normal and diseased human kidneys. Lab. Investig..

[7] Yamamoto T., Watanabe T., Ikegaya N., Fujigaki Y., Matsui K., Masaoka H., Nagase M., Hishida A. (1998). Expression of types I, II, and III TGF-β receptors in human glomerulonephritis. J. Am. Soc. Nephrol..

[8] Iwano M., Akai Y., Fujii Y., Dohi Y., Matsumura N., Dohi K. (1994). Intraglomerular expression of transforming growth factor-β1 (TGF-β1) mrna in patients with glomerulonephritis: Quantitative analysis by competitive polymerase chain reaction. Clin. Exp. Immunol..

[9] Yamamoto T., Noble N.A., Cohen A.H., Nast C.C., Hishida A., Gold L.I., Border W.A. (1996). Expression of transforming growth factor-β isoforms in human glomerular diseases. Kidney Int..

[10] Ebisawa T., Fukuchi M., Murakami G., Chiba T., Tanaka K., Imamura T., Miyazono K. (2001). Smurf1 interacts with transforming growth factor-β type I receptor through SMAD7 and induces receptor degradation. J. Biol. Chem..

[11] Yamamoto T., Nakamura T., Noble N.A., Ruoslahti E., Border W.A. (1993). Expression of transforming growth factor β is elevated in human and experimental diabetic nephropathy. Proc. Natl. Acad. Sci. USA.

[12] Yamamoto T., Noble N.A., Miller D.E., Border W.A. (1994). Sustained expression of TGF-β1 underlies development of progressive kidney fibrosis. Kidney Int..

[13] Sharma K., Ziyadeh F.N., Alzahabi B., McGowan T.A., Kapoor S., Kurnik B.R., Kurnik P.B., Weisberg L.S. (1997). Increased renal production of transforming growth factor-β1 in patients with type II diabetes. Diabetes.

[14] Terrell T.G., Working P.K., Chow C.P., Green J.D. (1993). Pathology of recombinant human transforming growth factor-β1 in rats and rabbits. Int. Rev. Exp. Pathol..

[15] Isaka Y., Fujiwara Y., Ueda N., Kaneda Y., Kamada T., Imai E. (1993). Glomerulosclerosis induced by in vivo transfection of transforming growth factor-β or platelet-derived growth factor gene into the rat kidney. J. Clin. Investig..

[16] Kopp J., Factor V., Mozes M., Nagy P., Sanderson N., Bottinger E., Klotman P., Thorgeirsson S. (1996). Transgenic mice with increased plasma levels of TGF-β1 develop progressive renal disease. Lab. Investig..

[17] Koesters R., Kaissling B., Lehir M., Picard N., Theilig F., Gebhardt R., Glick A.B., Hahnel B., Hosser H., Grone H.J. (2010). Tubular overexpression of transforming growth factor-β1 induces autophagy and fibrosis but not mesenchymal transition of renal epithelial cells. Am. J. Pathol..

[18] Border W.A., Okuda S., Languino L.R., Sporn M.B., Ruoslahti E. (1990). Suppression of experimental glomerulonephritis by antiserum against transforming growth factor-β1. Nature.

[19] Kasuga H., Ito Y., Sakamoto S., Kawachi H., Shimizu F., Yuzawa Y., Matsuo S. (2001). Effects of anti-TGF-β type II receptor antibody on experimental glomerulonephritis. Kidney Int..

[20] Akagi Y., Isaka Y., Arai M., Kaneko T., Takenaka M., Moriyama T., Kaneda Y., Ando A., Orita Y., Kamada T. (1996). Inhibition of TGF-β1 expression by antisense oligonucleotides suppressed extracellular matrix accumulation in experimental glomerulonephritis. Kidney Int..

[21] Chen S., Iglesias-de la Cruz M.C., Jim B., Hong S.W., Isono M., Ziyadeh F.N. (2003). Reversibility of established diabetic glomerulopathy by anti-TGF-β antibodies in *db*/*db* mice. Biochem. Biophys. Res. Commun..

[22] Ziyadeh F.N., Hoffman B.B., Han D.C., Iglesias-De La Cruz M.C., Hong S.W., Isono M., Chen S., McGowan T.A., Sharma K. (2000). Long-term prevention of renal insufficiency, excess matrix gene expression, and glomerular mesangial matrix expansion by treatment with monoclonal antitransforming growth factor-β antibody in *db*/*db* diabetic mice. Proc. Natl. Acad. Sci. USA.

[23] Miyajima A., Chen J., Lawrence C., Ledbetter S., Soslow R.A., Stern J., Jha S., Pigato J., Lemer M.L., Poppas D.P. (2000). Antibody to transforming growth factor-β ameliorates tubular apoptosis in unilateral ureteral obstruction. Kidney Int..

[24] Voelker J., Berg P.H., Sheetz M., Duffin K., Shen T., Moser B., Greene T., Blumenthal S.S., Rychlik I., Yagil Y. (2017). Anti-TGF-β1 antibody therapy in patients with diabetic nephropathy. J. Am. Soc. Nephrol..

[25] Vincenti F., Fervenza F.C., Campbell K.N., Diaz M., Gesualdo L., Nelson P., Praga M., Radhakrishnan J., Sellin L., Singh A. (2017). A phase 2, double-blind, placebo-controlled, randomized study of fresolimumab in patients with steroid-resistant primary focal segmental glomerulosclerosis. Kidney Int. Rep..

[26] Trachtman H., Fervenza F.C., Gipson D.S., Heering P., Jayne D.R., Peters H., Rota S., Remuzzi G., Rump L.C., Sellin L.K. (2011). A phase 1, single-dose study of fresolimumab, an anti-TGF-β antibody, in treatment-resistant primary focal segmental glomerulosclerosis. Kidney Int..

[27] McGaraughty S., Davis-Taber R.A., Zhu C.Z., Cole T.B., Nikkel A.L., Chhaya M., Doyle K.J., Olson L.M., Preston G.M., Grinnell C.M. (2017). Targeting anti-TGF-β therapy to fibrotic kidneys with a dual specificity antibody approach. J. Am. Soc. Nephrol..

[28] Iyer S.N., Gurujeyalakshmi G., Giri S.N. (1999). Effects of pirfenidone on procollagen gene expression at the transcriptional level in bleomycin hamster model of lung fibrosis. J. Pharmacol. Exp. Ther..

[29] Shihab F.S., Bennett W.M., Yi H., Andoh T.F. (2002). Pirfenidone treatment decreases transforming growth factor-β1 and matrix proteins and ameliorates fibrosis in chronic cyclosporine nephrotoxicity. Am. J. Transplant..

[30] Shimizu T., Fukagawa M., Kuroda T., Hata S., Iwasaki Y., Nemoto M., Shirai K., Yamauchi S., Margolin S.B., Shimizu F. (1997). Pirfenidone prevents collagen accumulation in the remnant kidney in rats with partial nephrectomy. Kidney Int..

[31] King T.E., Bradford W.Z., Castro-Bernardini S., Fagan E.A., Glaspole I., Glassberg M.K., Gorina E., Hopkins P.M., Kardatzke D., Lancaster L. (2014). A phase 3 trial of pirfenidone in patients with idiopathic pulmonary fibrosis. N. Eng. J. Med..

[32] Cho M.E., Smith D.C., Branton M.H., Penzak S.R., Kopp J.B. (2007). Pirfenidone slows renal function decline in patients with focal segmental glomerulosclerosis. Clin. J. Am. Soc. Nephrol..

[33] Sharma K., Ix J.H., Mathew A.V., Cho M., Pflueger A., Dunn S.R., Francos B., Sharma S., Falkner B., McGowan T.A. (2011). Pirfenidone for diabetic nephropathy. J. Am. Soc. Nephrol..

[34] Tsujie M., Isaka Y., Ando Y., Akagi Y., Kaneda Y., Ueda N., Imai E., Hori M. (2000). Gene transfer targeting interstitial fibroblasts by the artificial viral envelope-type hemagglutinating virus of japan liposome method. Kidney Int..

[35] Nakamura H., Isaka Y., Tsujie M., Rupprecht H.D., Akagi Y., Ueda N., Imai E., Hori M. (2002). Introduction of DNA enzyme for Egr-1 into tubulointerstitial fibroblasts by electroporation reduced interstitial α-smooth muscle actin expression and fibrosis in unilateral ureteral obstruction (UUO) rats. Gene Ther..

[36] Isaka Y., Nakamura H., Mizui M., Takabatake Y., Horio M., Kawachi H., Shimizu F., Imai E., Hori M. (2004). Dnazyme for TGF-β suppressed extracellular matrix accumulation in experimental glomerulonephritis. Kidney Int..

[37] Takabatake Y., Isaka Y., Mizui M., Kawachi H., Shimizu F., Ito T., Hori M., Imai E. (2005). Exploring rna interference as a therapeutic strategy for renal disease. Gene Ther..

[38] Isaka Y., Tsujie M., Ando Y., Nakamura H., Kaneda Y., Imai E., Hori M. (2000). Transforming growth factor-β1 antisense oligodeoxynucleotides block interstitial fibrosis in unilateral ureteral obstruction. Kidney Int..

[39] Watanabe S., Ogasawara T., Tamura Y., Saito T., Ikeda T., Suzuki N., Shimosawa T., Shibata S., Chung U.I., Nangaku M. (2017). Targeting gene expression to specific cells of kidney tubules in vivo, using adenoviral promoter fragments. PLoS ONE.

[40] Bogdahn U., Hau P., Stockhammer G., Venkataramana N.K., Mahapatra A.K., Suri A., Balasubramaniam A., Nair S., Oliushine V., Parfenov V. (2011). Targeted therapy for high-grade glioma with the TGF-β2 inhibitor trabedersen: Results of a randomized and controlled phase IIb study. Neuro-oncology.

[41] Annes J.P., Munger J.S., Rifkin D.B. (2003). Making sense of latent TGF-β activation. J. Cell Sci..

[42] Ma L.J., Yang H., Gaspert A., Carlesso G., Barty M.M., Davidson J.M., Sheppard D., Fogo A.B. (2003). Transforming growth factor-β-dependent and -independent pathways of induction of tubulointerstitial fibrosis in β6^−/−^ mice. Am. J. Pathol..

[43] Horan G.S., Wood S., Ona V., Li D.J., Lukashev M.E., Weinreb P.H., Simon K.J., Hahm K., Allaire N.E., Rinaldi N.J. (2008). Partial inhibition of integrin αvβ6 prevents pulmonary fibrosis without exacerbating inflammation. Am. J. Respir. Crit. Care Med..

[44] Yu L., Border W.A., Huang Y., Noble N.A. (2003). TGF-β isoform in renal fibrogenesis. Kidney Int..

[45] Derynck R., Zhang Y.E. (2003). Smad-dependent and Smad-independent pathways in TGF-β family signalling. Nature.

[46] Lin H.Y., Moustakas A., Knaus P., Wells R.G., Henis Y.I., Lodish H.F. (1995). The soluble exoplasmic domain of the type II transforming growth factor (TGF)-β receptor. A heterogeneously glycosylated protein with high affinity and selectivity for TGF-β ligands. J. Biol. Chem..

[47] Komesli S., Vivien D., Dutartre P. (1998). Chimeric extracellular domain type II transforming growth factor -β receptor fused to the Fc region of human immunoglobulin as a TGF-β antagonist. Eur. J. Biochem..

[48] Ren S., Babelova A., Moreth K., Xin C., Eberhardt W., Doller A., Pavenstädt H., Schaefer L., Pfeilschifter J., Huwiler A. (2009). Transforming growth factor-β2 upregulates sphingosine kinase-1 activity, which in turn attenuates the fibrotic response to TGF-β2 by impeding CTGF expression. Kidney Int..

[49] Isaka Y., Akagi Y., Ando Y., Tsujie M., Sudo T., Ohno N., Border W.A., Noble N.A., Kaneda Y., Hori M. (1999). Gene therapy by transforming growth factor-β receptor-IgG Fc chimera suppressed extracellular matrix accumulation in experimental glomerulonephritis. Kidney Int..

[50] Border W.A., Noble N.A., Yamamoto T., Harper J.R., Yamaguchi Y., Pierschbacher M.D., Ruoslahti E. (1992). Natural inhibitor of transforming growth factor-β protects against scarring in experimental kidney disease. Nature.

[51] Isaka Y., Brees D.K., Ikegaya K., Kaneda Y., Imai E., Noble N.A., Border W.A. (1996). Gene therapy by skeletal muscle expression of decorin prevents fibrotic disease in rat kidney. Nat. Med..

[52] Zeisberg M., Hanai J.-i., Sugimoto H., Mammoto T., Charytan D., Strutz F., Kalluri R. (2003). BMP-7 counteracts TGF-β1-induced epithelial-to-mesenchymal transition and reverses chronic renal injury. Nat. Med..

[53] Yue J., Mulder K.M. (2000). Activation of the mitogen-activated protein kinase pathway by transforming growth factor-β. Methods Mol. Biol..

[54] Kim S.I., Kwak J.H., Zachariah M., He Y., Wang L., Choi M.E. (2007). TGF-β-activated kinase 1 and TAK1-binding protein 1 cooperate to mediate TGF-β1-induced MKK3-p38 MAPK activation and stimulation of type I. collagen. Am. J. Physiol. Renal. Physiol..

[55] Sato M., Muragaki Y., Saika S., Roberts A.B., Ooshima A. (2003). Targeted disruption of TGF-β1/SMAD3 signaling protects against renal tubulointerstitial fibrosis induced by unilateral ureteral obstruction. J. Clin. Investig..

[56] Luangmonkong T., Suriguga S., Bigaeva E., Boersema M., Oosterhuis D., de Jong K.P., Schuppan D., Mutsaers H.A.M., Olinga P. (2017). Evaluating the antifibrotic potency of galunisertib in a human ex vivo model of liver fibrosis. Br. J. Pharmacol..

[57] Kavsak P., Rasmussen R.K., Causing C.G., Bonni S., Zhu H., Thomsen G.H., Wrana J.L. (2000). SMAD7 binds to Smurf2 to form an E3 ubiquitin ligase that targets the TGF-β receptor for degradation. Mol. Cell.

[58] Mu Y., Sundar R., Thakur N., Ekman M., Gudey S.K., Yakymovych M., Hermansson A., Dimitriou H., Bengoechea-Alonso M.T., Ericsson J. (2011). TRAF6 ubiquitinates TGF-β type I receptor to promote its cleavage and nuclear translocation in cancer. Nat. Commun..

[59] Gudey S.K., Sundar R., Mu Y., Wallenius A., Zang G., Bergh A., Heldin C.-H., Landström M. (2014). TRAF6 stimulates the tumor-promoting effects of TGF-β type I receptor through polyubiquitination and activation of presenilin 1. Sci. Signal..

[60] Arora K., Warrior R.A. (2001). A new Smurf in the village. Dev. Cell..

[61] Jin Y., Ratnam K., Chuang P.Y., Fan Y., Zhong Y., Dai Y., Mazloom A.R., Chen E.Y., D’Agati V., Xiong H. (2012). A systems approach identifies HIPK2 as a key regulator of kidney fibrosis. Nat. Med..

[62] Liu R., Das B., Xiao W., Li Z., Li H., Lee K., He J.C. (2017). A novel inhibitor of homeodomain interacting protein kinase 2 mitigates kidney fibrosis through inhibition of the TGF-β1/SMAD3 pathway. J. Am. Soc. Nephrol..

[63] Lin X., Duan X., Liang Y.Y., Su Y., Wrighton K.H., Long J., Hu M., Davis C.M., Wang J., Brunicardi F.C. (2016). PPM1A functions as a Smad phosphatase to terminate TGF-β signaling. Cell.

[64] Inoue K., Matsui I., Hamano T., Fujii N., Shimomura A., Nakano C., Kusunoki Y., Takabatake Y., Hirata M., Nishiyama A. (2012). Maxacalcitol ameliorates tubulointerstitial fibrosis in obstructed kidneys by recruiting PPM1A/VDR complex to pSMAD3. Lab. Investig..

[65] Meng X.M., Huang X.R., Xiao J., Chen H.Y., Zhong X., Chung A.C., Lan H.Y. (2012). Diverse roles of TGF-β receptor ii in renal fibrosis and inflammation in vivo and in vitro. J. Pathol..

[66] Connolly E.C., Freimuth J., Akhurst R.J. (2012). Complexities of TGF-β targeted cancer therapy. Int. J. Biol. Sci..

